# Supplementary material for: CASP1 Gene Polymorphisms and BAT1-NFKBIL-LTA-CASP1 Gene–Gene Interactions Are Associated with Restenosis after Coronary Stenting
Source: Biomolecules. 2022 May 31;12(6):765. doi: 10.3390/biom12060765 (PMC9221501; doi:10.3390/biom12060765)
Supplement: Supplementary file 1 [file biomolecules-12-00765-s001.zip › biomolecules-1720661-supplementary.pdf]

Supplementary Table S1. rs2239527 *C/G, NFKBIL1* rs2071592 *T/A, LTA* rs1800683 *G/A, CASP1* rs501192 *A/G,* and *CASP1* rs580253 *A/G* polymorphisms in CAD patients and healthy controls.

**Polymorphic site (rsID-number)**	**CAD *n* = 219 (*n* (%))**	**Controls *n* = 617 (n(%))**	***p***
rs2239527 *C/G*			
Allele			
C	278 (63.4)	810 (65.6)	NS
G	160 (36.5)	424 (34.3)	
Genotype			
CC	87 (39.7)	272 (44.1)	
CG	104 (47.4)	266 (43.1)	NS
GG	28 (12.8)	79 (12.8)	
rs2071592 *T/A*			
Allele			
T	281 (64.1)	811 (65.7)	NS
A	157 (35.8)	423 (34.2)	
Genotype			
TT	89 (40.6)	268 (43.4)	
TA	103 (47.0)	275 (44.6)	NS
AA	27 (12.3)	74 (12.0)	
rs1800683 *G/A*			
Allele			
G	275 (62.7)	794 (64.3)	NS
A	163 (37.2)	440 (35.6)	
Genotype			
GG	86 (39.2)	260 (42.1)	
GA	103 (47.0)	274 (44.4)	NS
AA	30 (13.7)	83 (13.5)	
rs501192 *G/A*			
Allele			
G	287 (65.5)	823 (67.5)	NS
A	151 (34.4)	395 (32.4)	
Genotype			
GG	101 (46.1)	277 (45.5)	
GA	85 (38.8)	269 (44.2)	0.040
AA	33 (15.0)	63 (10.3)	
rs580253 *A/G*			
Allele			
G	282 (64.4)	821 (67.4)	NS
A	156 (35.6)	397 (32.5)	
Genotype			
GG	99 (45.2)	275 (45.2)	
GA	84 (38.3)	271 (44.5)	0.011
AA	36 (16.4)	63 (10.3)	

Data are shown as n and frequency. *chi-square test.
